# Indicators of severe prognosis of scrub typhus: prognostic factors of scrub typhus severity

**DOI:** 10.1186/s12879-019-3903-9

**Published:** 2019-03-25

**Authors:** Hyun Lee Kim, Hye Rim Park, Choon-Mee Kim, Youn Jung Cha, Na Ra Yun, Dong-Min Kim

**Affiliations:** 1 Department of Internal Medicine, Chosun University College of Medicine, 588 Seosuk-dong, Dong-gu, Gwangju, 501-717 Republic of Korea; 2 Premedical sciences, College of Medicine, Chosun University, Gwang-ju, South Korea

**Keywords:** Scrub typhus, Prognosis, TNF-α level, APACHE II

## Abstract

**Background:**

Scrub typhus is an acute disease, characterized by symptoms of fever, which occurs due to infection by *Orientia tsutsugamushi*. In most cases, patients recover from the disease with appropriate treatment, but serious and fatal complications may occur. The present study examined laboratory findings and tumor necrosis factor-alpha (TNF-α) levels of scrub typhus patients to identify the prognostic predictors of disease severity.

**Method:**

Patients whose scrub typhus diagnosis was confirmed by elevated indirect fluorescent antibody (IFA) levels and positive polymerase chain reaction (PCR) results were classified according to disease severity into one of three groups; i.e., deceased (*n* = 7), severe (*n* = 15), and mild (n = 15) retrospectively registered. Additionally, the usefulness of modified Acute Physiology and Chronic Health Evaluation II (APACHE II) score, C-reactive protein (CRP) level, white blood cell (WBC) count, and TNF-α level as prognostic predictors were examined.

**Result:**

The mean TNF-α levels of the deceased, severe, and mild groups were 53.5 (range: 7.8–147.8), 26.0 (1.7–64.4), and 8.8 pg/mL (4.6–16.0), respectively. The results of Kruskal-Wallis tests showed statistically significant differences between the deceased and severe groups versus the mild group (*p* = 0.005). CRP level and Modified APACHE II score also differed significantly among the groups (*p* = 0.046 and 0.007, respectively); however, WBC count did not (*p* = 0.196).

**Conclusion:**

An elevated serum TNF-α level in patients with scrub typhus could predict a severe condition or death and may be useful in predicting patient prognosis.

## Background

Scrub typhus is an acute disease, characterized by symptoms of fever that occurs due to infection by *Orientia tsutsugamushi*, an obligate intracellular bacterium [1]. In most cases, the clinical course of scrub typhus is not severe and patients respond well to antibiotic therapy. The disease, however, sometimes manifests as pneumonia, acute renal failure, meningitis, upper gastrointestinal bleeding, or multiorgan failure, and patients may die due to such complications [2]. Several studies identified clinical markers including acute renal failure, hepatic dysfunction, central nervous system (CNS) dysfunction, abnormal chest X-ray, and hypotension as prognostic predictors of the severity of scrub typhus [3], but the current literature on laboratory findings and cytokine level as prognostic predictors is insufficient [4].

Human tumor necrosis factor-alpha (TNF-α, cachectin) is a nonglycosylated cytokine consisting of 157 amino acids mainly produced by activated macrophages; it is a pleiotropic molecule that plays important roles from inflammation to apoptosis [5]. Serum TNF-α levels were reportedly higher in patients with scrub typhus compared those in a normal control group [6], while another study showed a strong correlation between serum TNF-α level and disease severity in the acute phase of scrub typhus [7]. However, serum TNF-α levels in patients who die of scrub typhus have never been assessed. Moreover, data on the significance of TNF-α level as a prognostic predictor of the severity of scrub typhus is insufficient. Therefore, the present study investigated whether the laboratory findings and TNF-α level in patients with scrub typhus have significance as prognostic predictors of disease severity.

## Methods

This was a retrospective case-control study. Of the patients admitted to Chosun University Hospital and Haenam Hospital (a community branch hospital) for acute fever between 2004 and 2008, those clinically diagnosed with scrub typhus were enrolled in the study. To diagnose scrub typhus, serum antibodies were measured by indirect fluorescent antibody assay and nested polymerase chain reaction (PCR)-based test that amplifies a 56 kDa protein gene in buffy coat samples [7]. A serum antibody titer was repeatedly performed and a diagnosis of scrub typhus confirmed if the antibody level was elevated at least four-fold, or if the result of the blood buffy coat PCR was positive [8].

The severity of scrub typhus was classified according to admittance to the intensive care unit (ICU), hepatic involvement, respiratory and renal failures, and blood and neural involvement. Hepatic involvement was defined as an elevation of alanine aminotransferase (ALT) level over 100 U/L. Respiratory failure was defined as a reduction in arterial oxygen pressure below 70 mmHg and renal failure as an elevation of serum creatinine concentration over 1.8 mg/dL. Blood involvement was defined as a decreased platelet level below 150,000/μL, and neural involvement as a confused state of consciousness. Arterial blood gas analysis (ABGA) in the Acute Physiology and Chronic Health Evaluation II (APACHE II) to assess severity was not checked to some patients with mild disease prognosis; thus, the APACHE II scores which excluded the pH, PaO_2_, and A-a gradient which could be obtained through ABGA were referred to as modified APACHE II scores [9].

Patients were categorized into three groups according to disease severity, i.e., deceased, severe (patients admitted to the ICU or had the involvement of three or more organs), and mild (involvement of two or fewer organs). A total of seven patients died during the study period. To compare test results of deceased patients and survivors according to severity, an infectious disease physician randomly selected more than twice the numbers of severe and mild patients, respectively. Seven patients in the deceased group and 15 each in the severe and the mild groups with a confirmed diagnosis of scrub typhus were included in the final analysis.

Serum TNF-α levels were measured using a commercial enzyme-linked immunosorbent assay (ELISA) kit (Human TNF-alpha Quantikine HS ELISA, R&D systems) from Green Cross Laboratories. The minimal detected level of serum TNF-α was, on average, 0.106 pg/mL (range: 0.038–0.191). The study was approved by the Ethics in Human Research Committee of Chosun and Chonnam University Hospital (IRB-043-8).

Statistical analysis was performed using PASW Statistics for Windows, version 18.0 (SPSS Inc., Chicago, IL, USA), and the criterion for statistical significance was *P* = 0.05. Discrete variables were presented as percentages and continuous variables as means and ranges. To analyze differences in markers (TNF-α, CRP, WBC, and Modified APACHE II) between groups, nonparametric tests were performed using Mann-Whitney test and Kruskal-Wallis test. Correlations between markers were examined using Spearman coefficients and Wilcoxon signed-rank tests (nonparametric tests) were performed to examine differences in TNF-α level pre- and post-antibiotic use.

## Results

**Fig. 1:**
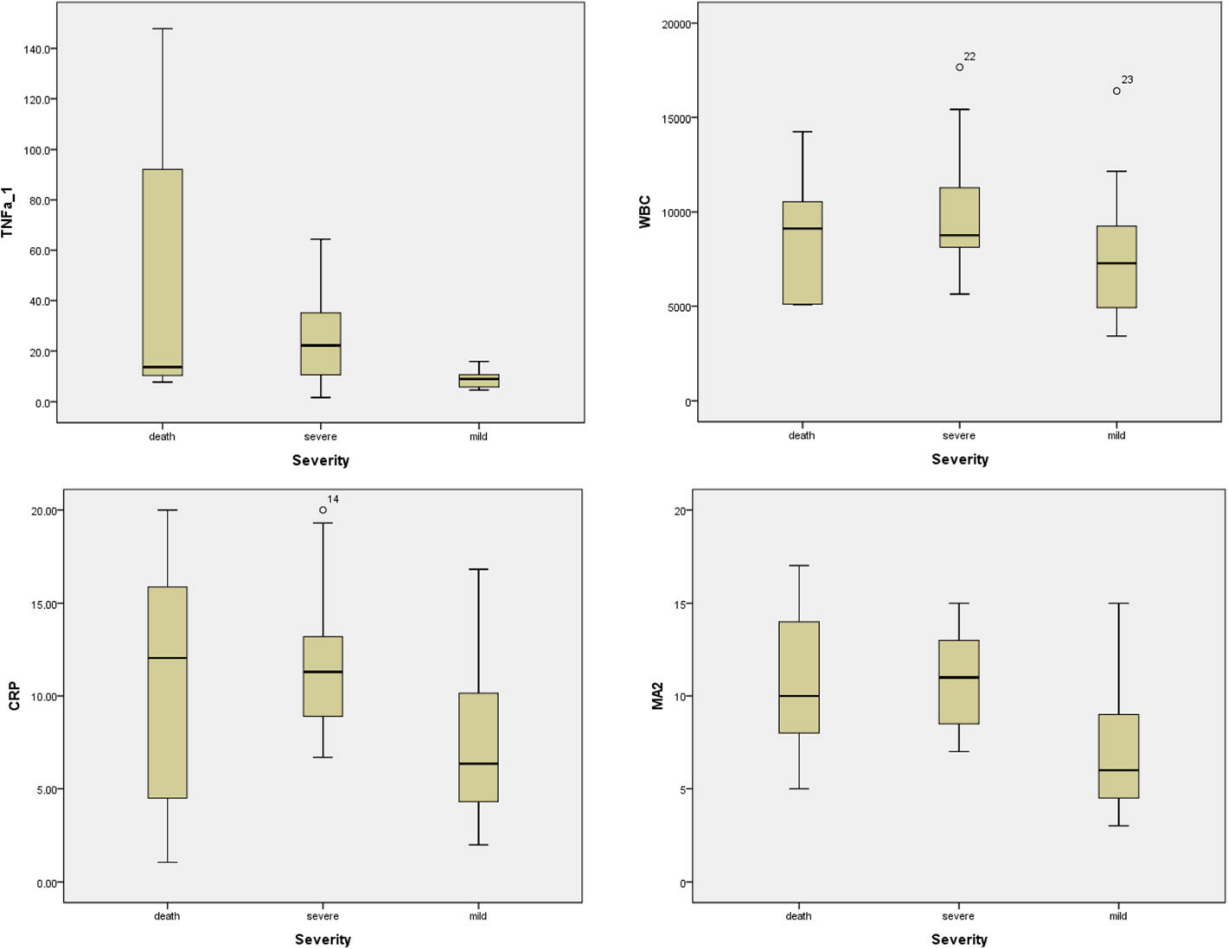
Comparison of the mean TNF-α level, CRP level, WBC count, and modified APACHE II score according to the severity of scrub typhus. The mean TNF-α levels were 53.5 (range: 7.8–147.8) 26.0 (1.7–64.4), and 8.8 pg/mL (4.6–16.0) in the deceased, severe, and mild groups, respectively. The mean WBC counts were 8539/mm^3^ (range: 5090-14,260/mm^3^), 10,048/mm^3^ (5640–17,660/mm^3^), and 7667/mm^3^ (3410–16,400/mm^3^) in deceased, severe, and mild groups, respectively. The mean CRP levels were 10.54 (range: 1.06–20.00), 12.06 (6.69–20.00), and 7.64 mg/dL (2.00–16.80) in the deceased, severe, and mild groups, respectively. The mean modified APACHE II scores were 10.86 (range: 5–17), 10.80 (7–15), and 6.87 (3–15) in the deceased, severe, and mild groups, respectively. Cf > TNF-α:tumor necrosis factor-alpha, WBC: White blood cell, CRP: C-reactive protein, MA2: Modified APACHE II

The mean patient age was 69.7 (37–89) years, with 18 males and 19 females. The mean TNF-α levels were 53.5 (range: 7.8–147.8), 26.0 (1.7–64.4), and 8.8 pg/mL (4.6–16.0) in the deceased, severe, and mild groups, respectively. The mean WBC counts were 8539/mm^3^ (5090-14,260/mm^3^), 10,048/mm^3^ (5640–17,660/mm^3^), and 7667/mm^3^ (3410–16,400/mm^3^) in the deceased, severe, and mild groups, respectively. The mean CRP levels were 10.54 (range: 1.06–20.00), 12.06 (6.69–20.00), and 7.64 mg/dL (2.00–16.80) in the deceased, severe, and mild groups, respectively. Finally, the mean modified APACHE II scores were 10.86 (range: 5–17), 10.80 (7–15), and 6.87 (3–15) in the deceased, severe, and mild groups, respectively (Fig. 1).

The Kruskal-Wallis test results showed statistically significant differences in TNF-α levels in the deceased, severe, and mild groups (*p* = 0.005). CRP level and modified APACHE II score also differed significantly among groups (*p* = 0.046 and 0.007, respectively), although WBC count did not (*p* = 0.196).

The results of the Mann-Whitney tests showed no differences between the deceased and severe groups with regards to TNF-α level, WBC count, CRP level, or modified APACHE II score; however, significant differences were observed in TNF-α level and modified APACHE II score (*p* = 0.014 and *p* = 0.032, respectively), but not WBC count and CRP level (*p* = 0.49 and *p* = 0.407, respectively) between the deceased and mild groups. Comparison of the severe and mild groups revealed significant differences in TNF-α level, modified APACHE II score, and CRP level (*p* = 0.002, p = 0.002, and *p* = 0.007), but not in WBC count (*p* = 0.07).

**Fig. 2:**
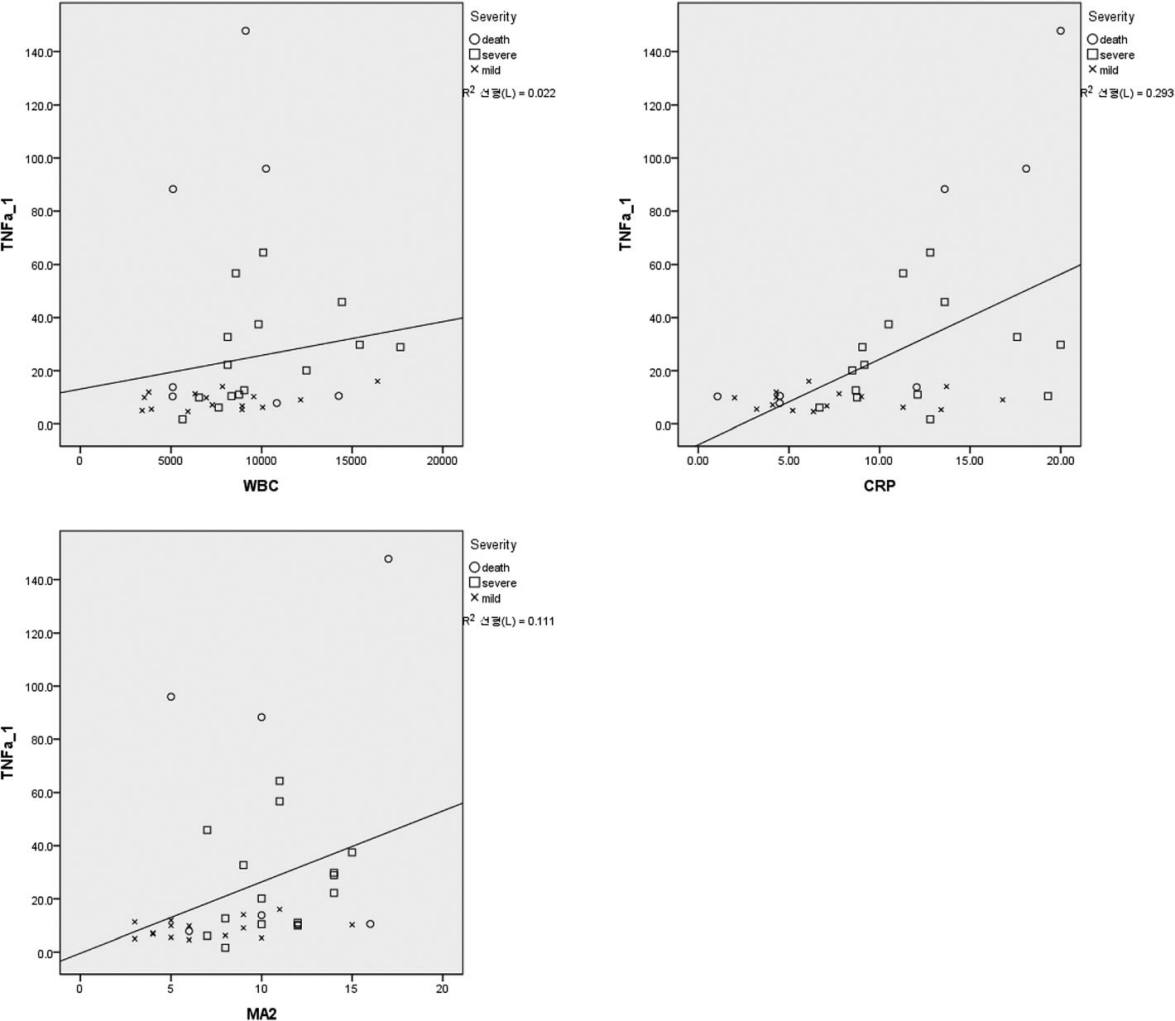
Correlations between CRP level, WBC, count modified APACHE II score, and TNF-α level in patients with scrub typhus. Cf > TNF-α:tumor necrosis factor-alpha, WBC: White blood cell, CRP: C-reactive protein, MA2: Modified APACHE II

**Fig. 3:**
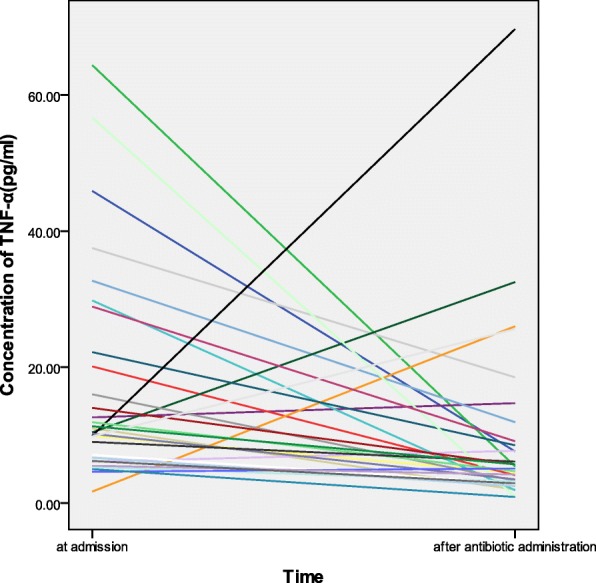
Serum TNF-α levels pre- and post-antibiotic use in patients with scrub typhus

TNF-α level was correlated with modified APACHE II score (Spearman coefficient = 0.467, *p* = 0.004), WBC count (Spearman coefficient = 0.398, *p* = 0.015), and CRP level (Spearman coefficient = 0.501, p = 0.002) (Fig. 2). Finally, TNF-α level was higher before antibiotic use than after antibiotic use (p = 0.01) (Fig. 3).

## Discussion

Scrub typhus is characterized by acute fever and often occurs during the fall in Korea. It can be easily treated if detected early, but patients can die from complications if treatment is delayed [8]. Accordingly, early detection and prompt prediction of disease severity and prognosis are important for appropriate treatment. The reported predictors of poor prognosis of scrub typhus include hypoalbuminemia (≤3.0 g/dL), absence of an eschar, old age, and coagulation disorder [2, 10]. Not only the virulence of scrub typhus causing agents but also imbalance or disorder in the host’s immune system can be important factors in the disease course; more specifically, cytokines may be involved in the mechanism of infection by *O. tsutsugamushi* [1]. Because the host’s immune response to bacterial infection affects the clinical course of the disease, the cytokines involved in the disease may be used to predict the disease severity in the patient. Indeed, several studies have reported that TNF-α plays critical roles in the mechanisms of various infectious diseases.^7^ TNF-α is an important cytokine that mediates diverse cellular immune responses with respect to infection and inflammation and is particularly known to play an important part in the host’s defense against a variety of intracellular pathogens [11].

A previous study reported that patients with scrub typhus had higher serum TNF-α concentrations compared to those of a normal control group [6]; similarly, another study observed a strong correlation between serum TNF-α level and disease severity in the acute phase of scrub typhus [7]. However, to our knowledge, no research had been conducted to examine the serum TNF-α concentration of patients who died of scrub typhus and research on other predictors of disease severity is insufficient. Therefore, the present study investigated the significance of serum TNF-α level as a predictor of disease severity in patients with scrub typhus, including deceased patients, and identified other predictors of disease severity.

Markers (TNF-α level, CRP level, WBC count, and modified APACHE II score) were compared across deceased patients and those with severe and mild scrub typhus. Serum TNF-α levels were higher in the deceased and severe groups compared to those in the mild group, while CRP level and modified APACHE II score were higher in the severe group. That is, elevated serum TNF-α, CRP level, and modified APACHE II score were associated with poor prognosis in patients with scrub typhus. In addition, serum TNF-α level was correlated with CRP level, modified APACHE II score, and WBC count. However, the difference in WBC count across the groups was not statistically significant.

The assessment of additional circulating molecules that may be associated with the increased TNF-α such as neutrophil related proteins or endothelial damage proteins associated with vascular damage, combining these with TNF- α would most likely increase the predictive values in scrub typhus patients. The limitations of the present study include the relatively small sample size (*n* = 37) and the lack of consideration of *O. tsutsugamushi* serotypes or genotypes. For adequate power to answer our research question, it need further study. To our knowledge, the present study is the first to compare TNF-α levels between patients who died of scrub typhus and those with mild disease, thus confirming the usefulness of TNF-α as a prognostic predictor.

## Conclusion

In conclusion, this study showed that an elevated serum TNF-α level can predict severe scrub typhus or death due to the disease, confirming that it may be useful for the prediction of the prognosis of scrub typhus patients.

## Abbreviations

APACHE II: Acute Physiology and Chronic Health Evaluation II
CNS: Central nervous system
CRP: C-reactive protein
IFA: Indirect fluorescent antibody
PCR: Polymerase chain reaction
TNF-α: Tumor necrosis factor-alpha
WBC: White blood cell
